# Prevalence of radiological findings among cases of severe secondary hyperparathyroidism

**DOI:** 10.1590/S1516-31802009000200004

**Published:** 2009-07-06

**Authors:** Paulo Gustavo Sampaio Lacativa, Felipe Malzac Franco, José Raimundo Pimentel, Pedro José de Mattos Patrício, Manoel Domingos da Cruz Gonçalves, Maria Lucia Fleiuss Farias

**Affiliations:** 1 MD. PhD student, Endocrinology Service, Department of Internal Medicine, Hospital Universitário Clementino Fraga Filho (HUCFF), Universidade Federal do Rio de Janeiro (UFRJ), Rio de Janeiro, Brazil.; 2 Scientific initiation student, Endocrinology Service, Department of Internal Medicine, Hospital Universitário Clementino Fraga Filho (HUCFF), Universidade Federal do Rio de Janeiro (UFRJ), Rio de Janeiro, Brazil.; 3 MD. Adjunct professor, Department of Radiology, Hospital Universitário Clementino Fraga Filho (HUCFF), Universidade Federal do Rio de Janeiro (UFRJ), Rio de Janeiro, Brazil.; 4 MD, MSc. Physician, Nephrology Service, Department of Internal Medicine, Hospital Universitário Clementino Fraga Filho (HUCFF), Universidade Federal do Rio de Janeiro (UFRJ), Rio de Janeiro, Brazil.; 5 PhD. Adjunct professor, Sector of Endocrine Surgery, Department of Surgery, Hospital Universitário Clementino Fraga Filho (HUCFF), Universidade Federal do Rio de Janeiro (UFRJ), Rio de Janeiro, Brazil.; 6 PhD. Adjunct professor, Endocrinology Service, Department of Internal Medicine, Hospital Universitário Clementino Fraga Filho (HUCFF), Universidade Federal do Rio de Janeiro (UFRJ), Rio de Janeiro, Brazil.

**Keywords:** Renal insufficiency chronic, Hyperparathyroidism, secondary, Calcinosis, Osteitis fibrosa cystica, Radiography, Insuficiência renal crônica, Hiperparatireoidismo secundário, Calcinose, Osteíte fibrosa cística, Radiografia

## Abstract

**CONTEXT AND OBJECTIVE:**

Patients with end stage renal disease (ESRD) and secondary hyperparathyroidism (HPT2) are prone to develop heterotopic calcifications and severe bone disease. Determination of the sites most commonly affected would decrease costs and patients’ exposure to X-ray radiation. The aim here was to determine which skeletal sites produce most radiographic findings, in order to evaluate hemodialysis patients with HPT2, and to describe the most prevalent radiographic findings.

**DESIGN AND SETTING:**

This study was cross-sectional, conducted in one center, the Hospital Universitário Clementino Fraga Filho (HUCFF), in Rio de Janeiro, Brazil.

**METHODS:**

Whole-body radiographs were obtained from 73 chronic hemodialysis patients with indications for parathyroidectomy due to severe HPT2. The regions studied were the skull, hands, wrists, clavicles, thoracic and lumbar column, long bones and pelvis. All the radiographs were analyzed by the same two radiologists, with great experience in bone disease interpretation.

**RESULTS:**

The most common abnormality was subperiosteal bone resorption, mostly at the phalanges and distal clavicles (94% of patients, each). “Rugger jersey spine” sign was found in 27%. Pathological fractures and deformities were seen in 27% and 33%, respectively. Calcifications were presented in 80%, mostly at the forearm fistula (42%), abdominal aorta and lower limb arteries (35% each). Brown tumors were present in 37% of the patients, mostly on the face and lower limbs (9% each).

**CONCLUSION:**

The greatest prevalence of bone findings were found on radiographs of the hands, wrists, lateral view of the thoracic and lumbar columns and femurs. The most prevalent findings were bone resorption and ectopic calcifications.

## RESUMO

**CONTEXTO E OBJETIVO:**

Pacientes com hiperparatireoidismo secundário (HPT2) à insuficiência renal crônica são propensos a desenvolver calcificações ectópicas e grave doença óssea. A determinação dos sítios mais revelantes pode diminuir o custo e a exposição do paciente a radiação desnecessária. O objetivo foi determinar quais locais radiológicos apresentam mais achados radiográficos para avaliar o HPT2 em pacientes em hemodiálise, assim como os achados mais prevalentes.

**TIPO DE ESTUDO E LOCAL:**

Estudo transversal, realizado no Hospital Universitário Clementino Fraga Filho (HUCFF), no Rio de Janeiro, Brasil.

**MÉTODOS:**

Radiografias de corpo inteiro foram obtidas de 73 pacientes em hemodiálise crônica que tiveram indicação de paratireoidectomia devido a HPT2 grave. As regiões estudadas foram crânio, mãos e punhos, clavículas, coluna torácica e lombar, ossos longos e pélvis. Todas as imagens foram analisadas pelos mesmos dois radiologistas, com grande experiência na interpretação de doenças do osso.

**RESULTADOS:**

A alteração mais comum foi reabsorção óssea subperiosteal, principalmente nas falanges distais e clavículas (ambos em 94% de pacientes). Sinal de “*rugger jersey*” foi descoberto em 27%. Fratura patológica e deformidades foram visualizadas em 27% e 33%, respectivamente. As calcificações foram encontradas em 80% dos pacientes, principalmente na fístula de antebraço (42%), aorta abdominal e artérias dos membros inferiores (ambos 35%). Tumores marrons estavam presentes em 37% dos pacientes, principalmente na face e nos membros inferiores (ambos 9%).

**CONCLUSÃO:**

As radiografias com achados mais prevalentes foram mãos e punhos, radiografia lateral da coluna torácica e lombar e fêmur. Os achados mais prevalentes foram reabsorção óssea e calcificação ectópica.

## INTRODUCTION

The term renal osteodystrophy represents a variety of bone disorders caused by chronic renal failure (CRF). The most common abnormalities are secondary hyperparathyroidism (HPT2) and osteitis fibrosa cystica, with extensive bone marrow fibrosis and increased osteoclastic bone resorption.^1^

Osteitis fibrosa cystica (OFC) is characterized by several types of radiographic findings. Bone resorption occurs because of increased osteoclastic activity and affects all bone surfaces at different skeletal sites. It may be subperiosteal, intracortical, endosteal, trabecular, subchondral, subligamentous or subtendinous.^2^ Subperiosteal bone resorption is the most characteristic radiographic feature of hyperparathyroidism and is found in the phalanges, humerus and distal epiphysis of the clavicles.^3^^,^^4^ When resorption is subchondral, like in the sacroiliac joints, it can mimic the widening of the pubic symphysis, leading to “pseudo-widening” of the joint.^5^ It occurs in different joints, particularly the sacroiliac, sternoclavicular and acromioclavicular joints.^2^ Intracortical and endosteal resorption can cause scalloped defects of the inner cortical contour. Association of trabecular resorption (which causes loss of definition) and granular texture leads to a “salt and pepper” appearance on the skull. Subligamentous and subtendinous bone resorption also occurs at many sites, such as the ischial tuberosities, femoral trochanters and insertions of the coracoclavicular ligaments.^2^ Losses of lamina dura of the teeth are also usually due to bone resorption.

Osteosclerosis can also occur in HPT2. Such changes are frequently found despite the presence and predominance of resorption. They are related either to excessive osteoblastic cell function in response to bone resorption, or to increased production of mineralized osteoid.^2^ Increased amounts of trabecular bone predominate in the axial skeleton, such as in the pelvis, ribs, spine and skull. One of the typical findings is broad osteosclerosis located below the endplates of the vertebral bodies, representing accumulations of excess osteoid, with normal density in the middle parts. This finding is called “rugger jersey spine sign”.^6^ Another typical example is sclerosis of the cortical surface of the cranium.

In severe cases of OFC, some bone deformities and fragility fractures may appear. Excessive resorption of the terminal phalanges may cause a deformity named acroosteolysis.^2^ Severe resorption in the sacroiliac joint may cause great damage to the pelvis, thus leading to deformities that can impair the ability to walk. Thoracic vertebral fractures increase the anteroposterior diameter and enlarge the base, and thus the thorax can take on a “bell mouth” shape. In cases of thoracic kyphoscoliosis, abnormal curvature and vertebral rotation may lead to chest deformity.

Fragility fractures sometimes occur at the sites of brown tumors. These lesions are caused by rapid osteoclastic activity and peritrabecular fibrosis, and are usually well-defined purely lytic lesions with the cortex thinned and expanded but not penetrated. Since they are usually painless, the clinical diagnosis is commonly made when the patient presents a fracture. However, a brown tumor may cause spinal cord compression when it involves the column, or it may cause breathing or eating difficulties when it deforms the face.^7^^,^^8^ Brown tumors appear mostly at the pelvis, ribs, clavicles, mandible and extremities.^8^

Metastatic calcification occurs when the calcium/phosphate solubility product in extracellular fluid is exceeded.^9^ Its presence has become a very important sign since Block et al. demonstrated the positive correlation between mortality risk and plasma calcium-phosphate product,^10^ especially due to vascular calcification, including in the coronary arteries. Other types of soft-tissue calcifications, such as visceral and periarticular calcifications, are also frequently seen in patients undergoing long-term hemodialysis.^11^^,^^12^ Tumoral calcinosis is the calcification of periarticular subcutaneous tissues around the major joints. Typically, the hips and shoulders are affected, although additional joints such as the elbows, feet, hands and wrists can become involved.^13^

Although bone histology remains the best method for differentiating between the forms of renal osteodystrophy, serum levels of parathyroid hormone (PTH) are commonly used for diagnosing secondary hyperparathyroidism and OFC.^14^ In patients with end stage renal disease (ESRD) and chronic hemodialysis, PTH serum levels are considered adequate between 150 and 300 pg/ml.^15^ Four to eightfold elevations of PTH are predictive for high-turnover bone disease and levels above this are strongly correlated with OFC.^16^^,^^17^

Since bone radiological abnormalities associated with ESRD often appear late, and because radiographic findings are less sensitive than PTH levels, use of radiographic images for screening purposes has been abandoned, and nowadays is reserved for symptomatic patients.^18^ However, in patients with very high levels of PTH, radiographs have some clinical indications, such as for ruling out dialytic amyloidosis.^19^ and for identifying several common complications of HPT2, such as skeletal lesions and heterotopic calcifications. Other important issues relating to X-ray images include: location of brown tumors, thus leading to actions for preventing fragility fractures; detection of osteosclerosis in vertebral bodies or calcification of abdominal vessels, in order to correctly interpret overestimated bone mineral density at such sites;^20^ findings of metastatic calcifications, thus requiring more rigid control over the calcium-phosphate product, including indication of parathyroidectomy;^19^ diagnosis of vertebral deformities and fractures, thus pointing towards better control over HPT2;^19^ and the possibility of predicting the severity of hungry bone syndrome (post-parathyroidectomy hypocalcemia) based on the severity of the changes to bone structures.^21^

Radiological HPT2 abnormalities are systemic, but it is not practical to evaluate all parts of the body, because of the costs and patient exposure to radiation. The present paper may help physicians to choose which sites would help most in evaluating hemodialysis patients with very high levels of PTH.

## OBJECTIVE

The aim here was to determine the most prevalent radiographic findings and to describe which skeletal sites produce them more often in hemodialysis patients with HPT2.

## METHODS AND PATIENTS

Patients with ESRD who had severe HPT2 were recruited from hemodialysis centers in the city of Rio de Janeiro, Brazil. They were studied at Hospital Universitário Clementino Fraga Filho from December 2001 to July 2006, in accordance with a protocol established by the Divisions of Endocrinology and Nephrology and the Department of Surgery of Universidade Federal do Rio de Janeiro.

All patients included in this study fulfilled the criteria used for indicating parathyroidectomy, i. e. serum PTH levels at or above ten times the upper limit of the normal range; non-response to oral medications (high doses of calcitriol plus calcium carbonate and phosphate binders) and one or more of the following: persistent hypercalcemia despite discontinuation of calcium and calcitriol; persistent symptomatic hypercalcemia following kidney transplantation; calcium-phosphorus product > 70 mg/dl;^2^ bone pain unresponsive to oral treatment; pathological fractures; bone deformities; ectopic calcifications; incapacitating arthritis or periarthritis; tendon rupture; untreatable pruritus; or brown tumors (in cases in which urgent surgical removal of the mass was needed).^19^

All 73 patients included in the analysis were dialyzed three times per week. There were 45 females (61.6%) and 28 males, and the mean age was 45.1 ± 11.6 years. With regard to ethnicity, 33 were black (45.2%), 21 were mixed (28.8%) and 19 were white (26.0%). The mean length of time on dialysis was 96.7 ± 46.1 months. The causes of CRF were: hypertensive nephrosclerosis in 41 patients (56.2%); glomerulonephritis in eight (10.9%); systemic lupus erythematosus in six (8.2%); obstructive nephropathies in five (6.8%); adult polycystic kidney disease in three (4.1%); interstitial nephritis in two (2.7%); chronic pyelonephritis in two (2.7%); renal tuberculosis in one (1.4%); diabetic nephropathies in one (1.4%); and unknown etiology in four (5.5%). None of these diseases were active, except for hypertension and diabetes; and no bone lesions relating to them were found. Serum calcium levels were corrected for serum albumin concentration, according to the formula:^22^ cCa (mg/dl) = sCa (mg/dl) + 0.8 [4.0 - SA (mg/dl)], where cCa = calcium corrected for albumin, sCa = serum calcium and SA = serum albumin.

The mean values of the biochemical parameters were: calcium (corrected for albumin) 10.32 ± 1.09 mg/dl (normal range: 8.5-10 .1<mg/ dl); phosphate 5.42 ± 1.61 mg/dl (normal range: 2.5 - 4.9 mg/dl); calcium-phosphate product 55.73 ± 19.45 (mg/dl);^2^ PTH: 2363.40 ± 1277.40 pg/ml (normal range: 7 - 53 pg/ml) and total alkaline phosphatase 838.00 ± 869.57 U/l (normal range: 50 - 136 U/l). No patient had serum aluminum greater than 60 µg/l.

Radiographs of the whole body were performed on all patients, which included the skull (lateral and anteroposterior views), hands and wrists (anteroposterior view), clavicles, thoracic and lumbar columns (lateral and anteroposterior views), long bones (radio, humerus, femur and tibia) and pelvis. All the radiographs were analyzed by two different radiologists who were experts on bone disease interpretation. A third opinion from another radiologist was used in cases in which the readings were discordant. The intraobserver variation for radiographs was estimated to be between 1.5 and 3.0% and the interobserver variation from 3.5 to 5.0% for the presence of each bone lesion.

The confidence interval for the prevalence of each bone lesion was calculated between 6.4 and 2.2%, with 95% of confidence interval. The minimum number of patients for the sample was calculated as 67 patients. It was considered that the mean prevalence of radiographic findings of secondary hyperparathyroidism would be 49.31%,^3^^,^^7^^,^^23^^,^^24^^,^^25^ with absolute precision of 10% and a significance level of 10%.

## RESULTS

The proportions of patients with severe HPT2 in whom radiographs detected bone resorption, osteosclerosis, bone deformities, brown tumors, fractures and ectopic calcifications are listed in Table 1.

**Figure 1.**
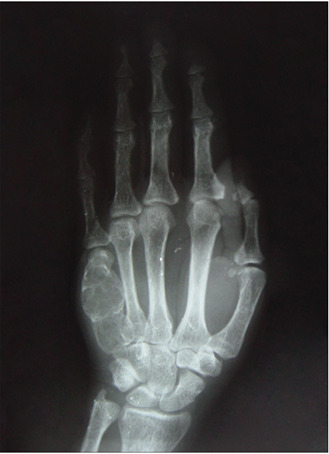
Hand radiograph showing subperiosteal bone resorption at the radial aspect of the middle phalanges, with loss of definition of the peripheral cortex; and a brown tumor, consisting of a cystic, expansible, well-defined lesion, with the cortex thinned and expanded but not penetrated, in the fifth metacarpal.

**Figure 2.**
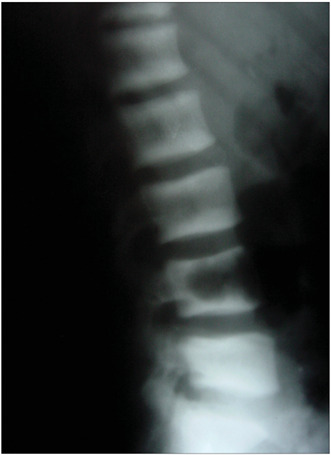
Lumbar column radiograph (lateral view) showing band-like regions of increased opacity (osteosclerosis) at the upper and lower margins, with normal density in the middle parts of the vertebral bodies, which is typical of the “rugger jersey spine” sign.

**Figure 3.**
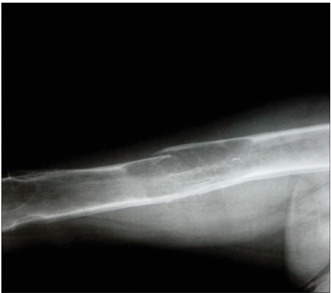
Femoral radiograph showing fragility fracture due to brown tumor in the femoral diaphysis; in the distal region of the femur, there are another two brown tumors, consisting of expansible well-defined lesions.

**Figure 4.**
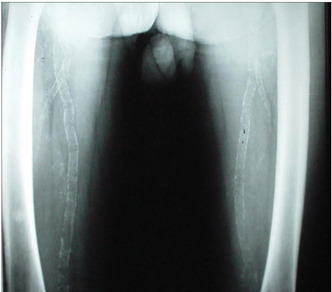
Femoral radiograph showing vascular calcifications of the femoral vessels.

In summary, all the 73 patients presented evidence of bone resorption on radiographs. The most prevalent types were subperiosteal resorption of the phalanges (Figure 1) and the distal ends of the clavicles, which were both present in 69 patients (94.5%), followed by “salt and pepper” skull patterns, diagnosed in 64 patients (87.7%). The lamina dura was absent in 46 patients, but another 14 patients did not have any teeth. “Pseudo-widening” of the sacroiliac joint was seen in 53 patients (72.6%). Osteosclerosis was also detected in these radiographs (78.1%), in the form of sclerosis of the cortical surface of the cranium in 54 patients (74.0%) and “rugger jersey spine sign” in 20 (27.4%) (Figure 2). Bone deformities were found in 27 patients (37.0%), mainly thoracic kyphoscoliosis (21.9% of all patients) and acroosteolysis (17.8% of all patients). Pelvic deformities were rare, occurring only in one patient (1.4%). Pathological fractures were found in 20 patients (27.4%), mostly in vertebral bodies (12.3%). Brown tumors were seen at several sites, including the face, lower and upper limbs (Figure 1 and 3), clavicles and ribs. In some patients, these lesions were the cause of the pathological fracture (Figure 3). Ectopic calcifications were very common; present in 80.8% of the patients. Vascular calcifications were found mostly in forearm fistulas in 33 patients (45.2%), lower limbs (32.9%) (Figure 4) and abdominal vessels (32.9%).

**Figure 5.**
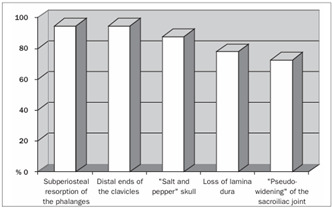
Presence of bone resorption according to bone sites.

**Figure 6.**
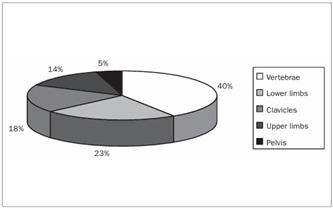
Distribution of fracture presence according to bone sites.

We also separately studied the sites of the skeleton at which specific signs of HPT2 could be detected on radiographs, as shown in Table 2, particularly with regard to bone resorption (Figure 5) and fractures (Figure 6). Although some specific abnormalities could be recognized at most sites, none of the sites presented all of the radiological HPT2 abnormalities.

Radiographs of the skull in lateral view demonstrated the typical finding of “salt and pepper” and loss of lamina dura in 69 patients, which means that bone resorption could be detected in 94.5% of the cases. Skull radiographs also made it possible to diagnose osteosclerosis in 94.7% of the patients, who presented sclerosis of the cortical surface of the cranium. Brown tumors are frequent in the maxillary and mandibular bones, and were detected in one third of the patients from the radiographs of these sites. Radiographs of the head also led to diagnosing of carotid calcification in 15.2% of the patients.

The radiographic evaluation of hands and wrists showed that 94.5% of patients presented bone resorption, due to subperiosteal resorption of the phalanges (Figure 1). Acroosteolysis accounted for 54.2% of all of the bone deformities, and one quarter of the brown tumors (Figure 1) could be demonstrated at this site. Vascular calcifications could be seen in more than half of the patients, especially when an arterial fistula was near the wrist.

Clavicular radiographs only showed bone resorption and a few brown tumors (22.2% of all the patients with this abnormality). The lateral view of the thorax detected bone deformities (91.7%) and fractures (20.0%), especially in the ribs. The lateral view of the lumbar spine could detect fractures due to vertebral deformities in 45.0% of the patients. Vascular calcification could also be diagnosed in 40.7% of the patients, due to abdominal aorta and iliac vessel calcifications.

The radiological evaluation of the pelvis demonstrated bone resorption due to “pseudo-widening” of the sacroiliac joint (72.6% of the cases), and also vascular calcifications in 15 patients (25.4% of all of the patients with this finding), but other abnormalities were only rarely seen: two patients had brown tumors, one had fractures and another had bone deformities.

The radiographs of the femurs were useful for detecting vascular calcifications (39%) (Figure 4), fractures (25%) and brown tumors (18.5%) (Figure 3). The tibia and humerus were sites with few findings.

**Table 1.** Number of patients (and percentages) for each secondary hyperparathyroidism (HPT2) bone finding

Location of radiological findings	n (%)
Bone resorption	73 (100.0%)
Subperiosteal resorption of the phalanges	69 (94.5%)
Distal ends of the clavicles	69 (94.5%)
“Salt and pepper” skull	64 (87.7%)
Loss of lamina dura (*)	46 (77.9%)
“Pseudo-widening” of the sacroiliac joint	53 (72.6%)
Osteosclerosis	57 (78.1%)
Sclerosis of the cortical surface of the cranium	54 (74.0%)
“Rugger jersey spine”	20 (27.4%)
Bone deformities	24 (32.9%)
Thoracic kyphoscoliosis	16 (21.9%)
Acroosteolysis	13 (17.8%)
Other thoracic deformities	6 (8.2%)
Pelvic deformities	1 (1.4%)
Fractures	20 (27.4%)
Vertebrae	9 (12.3%)
Lower limbs	5 (6.8%)
Clavicles	4 (5.5%)
Upper limbs	3 (4.1%)
Pelvis	1 (1.4%)
Brown tumor	27 (37.0%)
Face	9 (12.3%)
Lower limbs	7 (9.6%)
Upper limbs	7 (9.6%)
Clavicles	6 (8.2%)
Ribs	5 (6.8%)
Pelvis	2 (2.7%)
Vertebrae	1 (1.4%)
Ectopic calcification	59 (80.8%)
Subcutaneous calcification	18 (24.7%)
Vascular calcification	54 (74.0%)
Fistula	33 (45.2%)
Lower limbs	24 (32.9%)
Abdominal aortic/iliac	24 (32.9%)
Pelvis	15 (20.5%)
Upper limbs	14 (19.2%)
Neck	9 (12.3%)

*Fourteen patients did not have any teeth, so the percentage for the lamina dura was calculated in relation to a total of 59 patients.

**Table 2.** Patients (n and %) that presented with each secondary hyperparathyroidism (HPT2) bone finding, according to different sites evaluated

HPT2 bone finding	Bone resorption	Osteosclerosis	Bone deformities	Fractures	Brown tumor	Vascular calcification
Sites						
Cranium (lateral view)	69 (94.5%)	54 (94.7%)	0	0	9 (33.3%)	9 (15.2%)
Hands and wrists	69 (94.5%)	0	13 (54.2%)	1 (5.0%)	7 (25.9%)	30 (50.8%)
Clavicle	69 (94.5%)	0	0	0	6 (22.2%)	0
Thoracic column (lateral view)	0	0	22 (91.7%)	4 (20.0%)	5 (18.5%)	2 (3.4%)
Lumbar column (lateral view)	0	20 (35.1%)	0	9 (45.0%)	1 (3.7%)	24 (40.7%)
Humerus	0	0	0	2 (10.0%)	2 (7.4%)	6 (10.2%)
Pelvis	53 (72.6%)	0	1 (4.1%)	1 (5.0%)	2 (7.4%)	15 (25.4%)
Femur	0	0	0	5 (25.0%)	5 (18.5%)	23 (39.0%)
Tibia	0	0	0	0	2 (7.4%)	6 (10.2%)
Total number of patients	73	57	24	20	27	59

## DISCUSSION

All of the patients studied presented severe forms of HPT2. For the purposes of this study, this feature of the sample was desired, so that bone changes would be more prevalent and easier to detect. On the other hand, the limitation of the conclusions is that they may not be applicable to mild forms of HPT2 or all populations of CRF cases. In these populations, radiographs are usually indicated with the purpose of differential diagnosis, but their usefulness is limited, since serum PTH is much more sensitive.^18^ The present study may, however, guide physicians towards selecting the most important regions to be studied, in order to detect specific signs of HPT2 and aid in managing patients with ESRD and very high levels of serum PTH.

Bone resorption detection is useful for differential diagnosis purposes.^19^ Subperiosteal erosion is first noted at the phalangeal tufts and in the radial aspects of the middle phalanges of the second and third fingers. Hand radiographs are useful not only for early radiological diagnosis, but also for monitoring the effect of treatment for HPT2.^2^^,^^18^ Since subperiosteal resorption of the phalanges is considered to be the most sensitive radiographic sign of osteitis fibrosa,^23^ its absence would rule out bone resorption at other frequently affected sites.^2^^,^^21^ In the present study, although the prevalence of subperiosteal resorption of the phalanges was not 100%, this finding had very high sensitivity (94.5%), as did findings from the clavicles and skull.

The “rugger jersey” spine sign is almost diagnostic of osteosclerosis associated with HPT2,^6^ and so this finding has differential diagnosis purposes. The problem is the very low prevalence of this sign, since osteosclerosis occurs in only about 20% of patients with renal osteodystrophy.^24^ In the present study, the lateral view of the lumbar column was able to detect “rugger jersey spine” sign in 27.4% of the patients, while cranial radiographs were able to detect osteosclerosis in 94.7% of the patients. Despite very high prevalence, sclerosis of the cranium is not specific for HPT2 and does not cause complications for patients: for these reasons, cranial radiographs are not useful. On the other hand, the finding of osteosclerosis in vertebral bodies is useful in clinical practice, since it may interfere with bone densitometry at this site.

The presence of skeletal deformities is very important, since it is a sign of unquestionable severity of HPT2 and a classical indication for parathyroidectomy^19^ due to complications such as bone pain, respiratory disorders and walking disabilities. The lateral view of the thoracic column was the best plane for detecting thoracic deformities on radiographs, but these deformities are easily detected in physical examinations. Since acroosteolysis may only be detected on hand radiographs, this view is very useful.

Detection of pathological fractures is also crucial, since they are a marker for severity of OFC, which is a classical indication for parathyroidectomy. They may cause several complications, including inability to walk, dependence on other people for basic tasks (such as eating or getting dressed) and increased mortality. Vertebral fractures are associated not only with increased morbidity, such as height loss, kyphosis, back pain, functional impairment and depression,^26^^,^^27^ but also with increased risk of hospitalization^28^ and relative risk of mortality,^29^^,^^30^^,^^31^ which may be almost nine times greater.^32^ Fractures that occur in the appendicular skeleton are usually clinical, and therefore radiographic evaluation has limited usefulness for diagnosing this complication. On the other hand, only about one in four vertebral fractures is clinically recognized.^33^ For this reason, the lateral view of the lumbar column is useful for evaluating this complication. In the present study, fractures were mostly observed in vertebrae, and all of them were in the lumbar vertebrae. Thus, spinal radiographs were able to detected 45% of the patients who presented fractures.

Brown tumors are considered to be the most pathognomonic of the many skeletal changes that accompany HPT2.^34^ The incidence of skeletal brown tumors in patients with CRF ranges from 1.5 to 13%.^7^^,^^25^ In the present study, the patients presented very severe HPT2 and the prevalence of these lesions was higher (32.9%). Brown tumors are usually painless and need no specific treatment in most cases, but they may cause pathological fractures. Their presence in the spine may lead to complications such as spinal cord compression.^7^ When they affect the face, they can cause disfiguring deformities and difficulties in breathing through the nose or eating. In these cases, they are very large and can easily be detected by physical examination.^8^ In the present study, brown tumors were most located on the face and limbs, and especially on the hands.

Ectopic calcification is a very important sign because of its prevalence and its relationship with mortality. At autopsy, metastatic calcifications have been detected in 60-80% of patients who had undergone dialytic therapy.^3^ Calcifications within the vessels walls affect central large and peripheral small arteries, and they appear as circular or linear radiodensities.^2^ The complications are vascular obstruction with consequent ischemia of the soft tissues, and they are risk factors for cardiovascular disease.^10^ Other related problems are fistula calcifications, which may cause complications through loss of functionality, and abdominal vessels calcifications, which may change the results from bone densitometry.^20^ In the present study, the very high prevalence of ectopic calcifications was of great concern (80.8%). Vascular calcifications were more prevalent in the hands (especially if there was a wrist fistula), lumbar column, pelvis and femur.

Hand radiographs probably remain the most commonly requested type of radiograph,^19^ since they can detect several typical changes in bone structure in severe forms of OFC.^35^ The radiographs of hands and wrists and pelvic radiographs were the ones associated with most findings of OFC in the present study. However, hands and wrists seem to be more important, since these sites allow detection of bone resorption, deformities (acroosteolysis), brown tumors and vascular calcification.

Skull evaluations detected some of the radiological findings of OFC, but had some clinical limitations. They could detect bone resorption in the same proportions but not better than radiographs of the hand, wrist and clavicles. The skull was also the site at which the presence of osteosclerosis (94.7%) and brown tumors (33.3%) were diagnosed most. However, these findings do not help the physician, since sclerosis of the cortical surface of the cranium does not cause any complications for patients, and brown tumors in the skull are unrelated to fractures. The disadvantage of evaluating this site is the inability to detect bone deformities and fractures.

Clavicular radiographs were useful for detecting bone resorption, although again, not better than hand radiographs. They also detected brown tumors in 22.2% of the patients. However, they were unable to detect other bone lesions due to HPT2.

Thoracic and lumbar column radiographs were very useful for detecting “rugger jersey spine” sign (35.1%), thoracic deformities (91.7%), brown tumors (22.2%), fractures (60.0%) and vascular calcification (51.1%). Although thoracic deformities can be recognized in physical examinations, the very high prevalence of other important findings makes it important to evaluate the thoracic and lumbar spine.

Pelvic radiographs showed most of the bone findings of HPT2, but with very low prevalence. Even the important findings of fracture and deformity can be detected on physical examination. Pelvic radiographs may be useful for detecting vascular calcification, but are not better than radiographs of hands and wrists, lumbar column and femurs. The same applies to humeral and tibial radiographs.

Femoral radiographs are very important for showing brown tumors, which in some cases cause fragility fractures, which are also detected at this site. Vascular calcifications are also frequently seen in femurs. Therefore, this location should also be evaluated in hemodialysis patients with very high levels of PTH.

## CONCLUSIONS

In hemodialysis patients with severe forms of HPT2, the most prevalent findings were bone resorption and ectopic calcifications. The radiographs with highest prevalence of bone findings were those on hands and wrists, with the ability to serve as differential diagnosis, for detection of bone deformities, brown tumors and vascular calcifications (especially in fistulas). Radiographs showing the lateral view of the thoracic and lumbar column were also useful, mainly because of detection of fragility fractures that may go unrecognized in physical examinations (in vertebrae and ribs) and the “rugger jersey sign”, which is a lesion very specific to HPT2 and vascular calcifications. Femoral radiographs were useful especially for detecting brown tumors that may cause fragility fractures and vascular calcifications. Pelvic and cranial radiographs, although very useful for detecting bone abnormalities of HPT2, did not add any more information for HPT2 evaluation.
